# A Case of Hepatic Encephalopathy With Diffuse Brainstem Signal Changes on Magnetic Resonance Imaging

**DOI:** 10.7759/cureus.81217

**Published:** 2025-03-26

**Authors:** Hiromu Yurimoto, Taiki Matsubayashi, Isamu Shibata, Misako Furuki, Masato Obayashi

**Affiliations:** 1 Neurology, National Hospital Organization Disaster Medical Center, Tokyo, JPN; 2 Gastroenterology, National Hospital Organization Disaster Medical Center, Tokyo, JPN

**Keywords:** apparent diffusion coefficient, brainstem, diffusion weighted imaging, hepatic encephalopathy, magnetic resonance imaging

## Abstract

Signal changes on brain MRI have been reported in hepatic encephalopathy; however, no specific findings have been established. Moreover, cases of hepatic encephalopathy presenting with MRI signal changes confined to the brainstem are rare.

A 75-year-old woman was admitted to our hospital with a one-day history of impaired consciousness. Blood tests revealed elevated ammonia and gamma-glutamyl transpeptidase levels, along with positive anti-mitochondrial M2 antibodies. Brain MRI on admission demonstrated diffuse and symmetrical hyperintensity in the midbrain and pons on fluid-attenuated inversion recovery (FLAIR) imaging, with corresponding hyperintensity on diffusion-weighted imaging (DWI) and low values on the apparent diffusion coefficient (ADC) map. No signal abnormalities were observed in the globus pallidus on T1-weighted imaging (T1WI). Whole-body CT revealed an irregular hepatic surface, a blunt liver edge, and splenomegaly, leading to a diagnosis of liver cirrhosis. Primary biliary cholangitis was confirmed based on serological findings. Following the initiation of branched-chain amino acid therapy, the patient’s consciousness improved by day 4 after admission, and the brainstem abnormalities on MRI resolved by day 10. She was ultimately diagnosed with hepatic encephalopathy.

This case highlights the importance of considering hepatic encephalopathy in the differential diagnosis of diffuse brainstem signal changes on MRI. The observed MRI features, including symmetrical signal changes, hyperintensity on DWI, and reversible imaging findings during the clinical course, support the diagnosis of metabolic encephalopathy. Additionally, the presence of hyperintensity on DWI with low ADC values may reflect an acute clinical course in hepatic encephalopathy. Furthermore, brainstem-limited signal changes on MRI could be associated with a favorable prognosis.

## Introduction

Hepatic encephalopathy occurs in 30-50% of cirrhotic patients undergoing transjugular intrahepatic portosystemic shunting [1]. Clinically, it presents with altered consciousness and various neuropsychiatric symptoms, including asterixis, delirium, convulsions, and coma [1]. The condition arises due to hepatic dysfunction, which impairs ammonia detoxification via the urea cycle, leading to elevated blood ammonia levels and disrupted neurotransmission in the brain [2]. Typically, compensatory mechanisms are activated to mitigate hyperammonemia; however, conditions such as gastrointestinal bleeding, infection, and constipation can precipitate an acute rise in blood ammonia levels, overwhelming these compensatory pathways [3].

Since hepatic encephalopathy lacks established diagnostic criteria, its diagnosis relies on a comprehensive assessment of clinical factors such as liver cirrhosis, clinical history, and elevated blood ammonia levels [4]. However, grading systems such as the West Haven criteria and the Hepatic Encephalopathy Scoring Algorithm have been proposed to classify its severity [5].

Brain MRI findings in hepatic encephalopathy often include hyperintensity in the globus pallidus on T1-weighted imaging (T1WI), though this is not specific and is also seen in chronic liver injury [6,7]. Other reported findings include hyperintensity along the white matter or corticospinal tract on fluid-attenuated inversion recovery (FLAIR) or T2-weighted imaging (T2WI) and diffuse cortical edema, particularly symmetrically involving the cingulate gyrus and insular cortex [8]. However, definitive MRI markers for hepatic encephalopathy remain unestablished, and cases with isolated brainstem signal changes are rarely recognized.

Here, we present a case of hepatic encephalopathy with a favorable prognosis, characterized by diffuse yet reversible symmetric brainstem signal changes on MRI. This case underscores the importance of considering hepatic encephalopathy in such MRI patterns and suggests that brainstem-limited changes may be associated with a better prognosis.

## Case presentation

A 75-year-old woman presented with black stools two days prior and a one-day history of cognitive impairment and decreased consciousness. She was brought to our hospital due to persistent impaired consciousness. Her medical history included postoperative breast cancer with bilateral mastectomy 10 years earlier, hypertension, cellulitis, stasis dermatitis, knee osteoarthritis, and lumbar spondylosis. She had no history of alcohol consumption. On admission, she exhibited a corpulent body, with a height of 159 cm, weight of 120 kg, and BMI of 47.5 kg/m^2^. Physical examination revealed as follows: body temperature, 37.4°C; blood pressure, 165/95 mmHg, heart rate, 76 beats/min; respiratory rate, 16 breaths/min; and oxygen saturation, 100 % on 3 L/min supplemental oxygen. A large amount of tarry stools was observed. Neurological examination revealed impaired consciousness with a Glasgow Coma Scale score of E1V1M4. The light reflex was prompt bilaterally, indicating preserved brainstem reflexes. No motor paralysis or involuntary movements, including convulsions, were noted.

Laboratory findings revealed mild thrombocytopenia, decreased prothrombin time, low albumin levels, and markedly elevated blood ammonia levels (210 µmol/L; reference range: 9-47 µmol/L). An elevated gamma-glutamyl transpeptidase level (56 U/L) was observed. There was no deficiency of vitamin B1 or hyponatremia. Immunological tests revealed positive results for the anti-mitochondrial M2 antibody (39.5 U/mL) and anti-nuclear antibody with speckled pattern and elevated results for immunoglobulin M and mac-2 binding protein glycosylation isomer. A cerebrospinal fluid (CSF) examination showed normal white blood cell count (6/3 µL), slightly elevated protein level (77.4 mg/dL), and normal glucose level. The normal CSF findings, combined with elevated ammonia levels, suggest that the altered consciousness was more likely due to metabolic encephalopathy, including hepatic encephalopathy, rather than meningitis or encephalitis. Laboratory results are summarized in Table 1.

**Table 1:** Laboratory parameters analyzed in the serum and CSF CSF: Cerebrospinal fluid; HBs: Hepatitis B virus surface; HCV: Hepatitis C antibodies

	Laboratory parameters	Value (units)	Reference value
Ser	White blood cell	1,1000/μL	4,000-10,000
	Neutrophils	64.0%	40-70
	Hemoglobin	11.6 g/dL	11.6-14.8
	Platelet count	12.7 × 10⁴/μL	15.8-34.8 × 10⁴
	Prothrombin time	63%	70-130
	Albumin	3.3 g/dL	4.1-5.1
	Aspartate aminotransferase	29 U/L	13-30
	Alanine aminotransferase	15 U/L	7-23
	Alkaline phosphatase	35 U/L	38-113
	Gamma-glutamyl transpeptidase	56 U/L	9-32
	C-Reactive Protein	0.09 mg/dL	<0.5
	HBs antigen	Negative	Negative
	Anti-HCV antibodies	Negative	Negative
	Ammonia	210 µmol/L	9-47
	Vitamin B1	156 ng/mL	24-66
	Glucose	99 mg/dL	73-110
	Anti-mitochondrial M2 antibody	39.5 U/mL	<7.0
	Anti-nuclear antibody	1:320	<1:40
	Immunoglobulin M	348 mg/dL	50-269
	Mac-2 binding protein glycosylation isomer	2.82	<1.0
CSF	Color	Clear	-
	White blood cell	6/3 μL	0-15
	Lymphocytes	83%	40-80
	Protein	77.4 mg/dL	15-45
	Glucose	59 mg/dL	50-70

**Figure 1:**
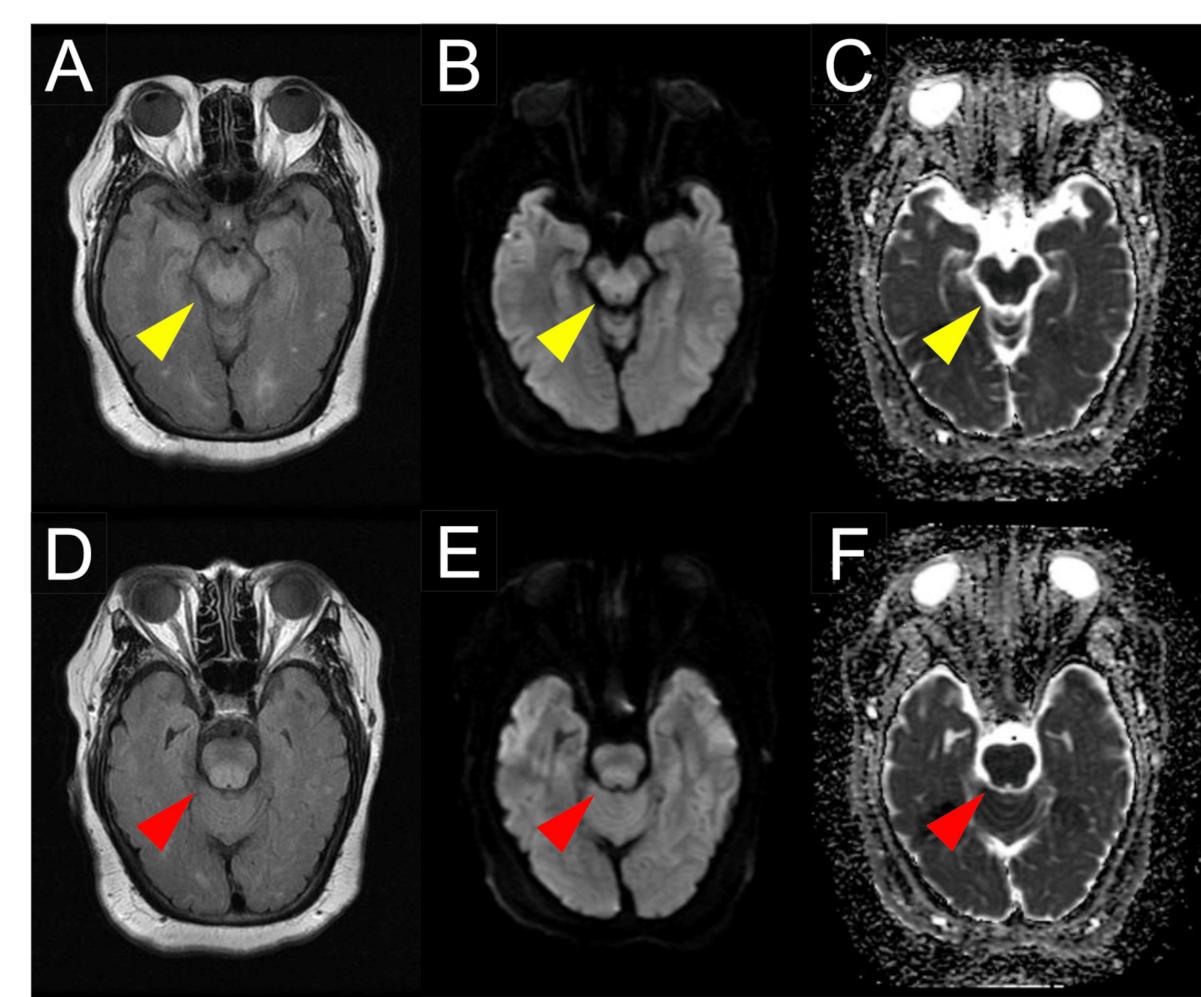
MRI findings on admission MRI showed hyperintensity in the midbrain tegmentum, sparing the cerebral crus, on FLAIR imaging (A), along with hyperintensity on DWI (B) and low values on the ADC map (C) (yellow arrowheads). Additionally, MRI revealed hyperintensity in the pontine tegmentum on FLAIR (D), with corresponding hyperintensity on DWI (E) and low values on the ADC map (F) (red arrowheads). ADC: Apparent diffusion coefficient; DWI: Diffusion-weighted imaging; FLAIR: Fluid-attenuated inversion recovery

Non-contrast whole-body CT showed an irregular hepatic surface, a blunt liver edge, and splenomegaly, with no evidence of ascites. Findings suggestive of malignancy were not detected on whole-body CT. Brain MRI demonstrated diffuse hyperintensity signals in the brainstem extending from the midbrain to the pons on FLAIR imaging, all symmetrically distributed (Figure 1). The brainstem lesions appeared as hyperintensities on diffusion-weighted imaging (DWI) with corresponding low values on the apparent diffusion coefficient (ADC) map (Figure 1). The symmetrical high signal intensity observed in the medial temporal lobes on DWI and FLAIR was determined to be an artifact. No hyperintensity was observed in the thalamus. The globus pallidus did not exhibit hyperintensity signals on T1WI.

**Figure 2:**
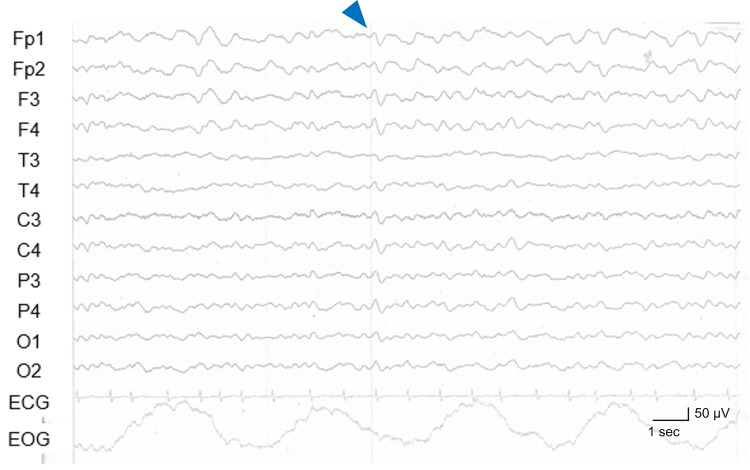
EEG findings on day 4 of admission Slow-wave activity with a basic rhythm of 5-6 Hz and occasional bilaterally synchronous triphasic waves (blue arrowhead) were observed. EEG: Electroencephalogram

An electroencephalogram (EEG) performed on day 4 after the admission demonstrated slow-wave activity with a basic rhythm of 5-6 Hz and occasional bilaterally synchronous triphasic waves (Figure 2).

Based on the CT findings and laboratory results showing elevated gamma-glutamyl transpeptidase levels and the presence of anti-mitochondrial M2 antibodies, she was diagnosed with liver cirrhosis due to primary biliary cholangitis [9]. The Child-Pugh score was 9 points, corresponding to grade B.

**Figure 3:**
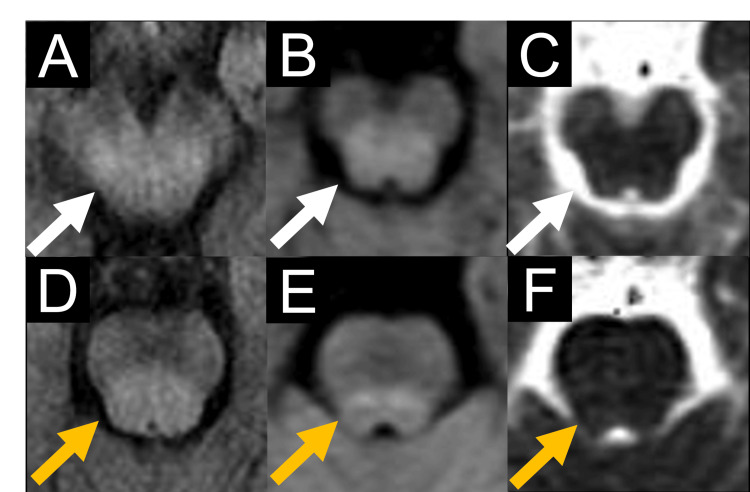
MRI findings on day 3 of admission MRI showed remained hyperintensity in the midbrain tegmentum, sparing the cerebral crus, on FLAIR imaging (A), along with hyperintensity on DWI (B) and low values on the ADC map (C) (white arrows). Furthermore, MRI revealed hyperintensity in the pontine tegmentum on FLAIR (D), with corresponding hyperintensity on DWI (E) and low values on the ADC map (F) (orange arrows). ADC: Apparent diffusion coefficient; DWI: Diffusion-weighted imaging; FLAIR: Fluid-attenuated inversion recovery

**Figure 4:**
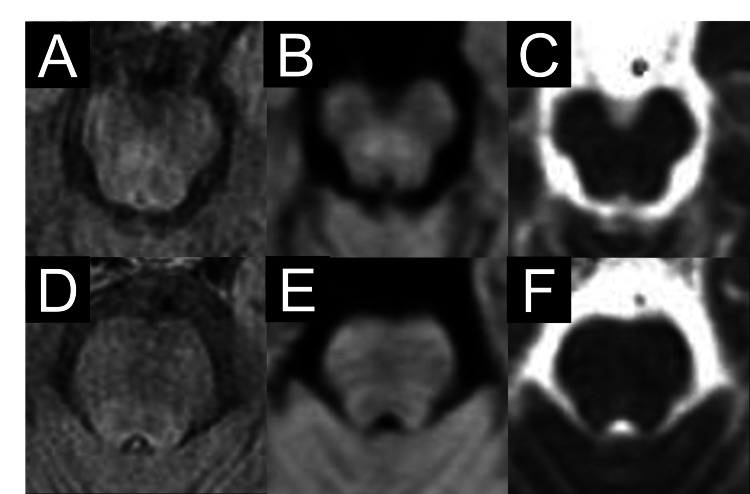
MRI findings on day 10 of admission Hyperintensity in the midbrain tegmentum, sparing the cerebral crus, resolved on FLAIR imaging (A), DWI (B), and the ADC map (C). Similarly, hyperintensity in the pontine tegmentum resolved on FLAIR (D), with corresponding resolution of hyperintensity on DWI (E) and low values on the ADC map (F). ADC: Apparent diffusion coefficient; DWI: Diffusion-weighted imaging; FLAIR: Fluid-attenuated inversion recovery

For the treatment of impaired consciousness, intravenous branched-chain amino acids were initiated promptly upon admission due to suspected hepatic encephalopathy. Additionally, acyclovir (1,500 mg/day) and ceftriaxone (2 g/day) were administered simultaneously to address the possibility of an underlying infection. Her consciousness gradually improved after admission, becoming clear by day 4 after admission. She was diagnosed with hepatic encephalopathy based on clinical, laboratory, and imaging findings. Rapid clinical improvement of her altered consciousness with branched-chain amino acid therapy and elevated ammonia levels on laboratory tests were consistent with hepatic encephalopathy. EEG findings of slow waves and triphasic waves, along with the disappearance of brainstem signal changes on MRI following neurological recovery, further supported this diagnosis. Additionally, primary biliary cholangitis with cirrhosis was a plausible underlying condition predisposing her to hepatic encephalopathy. This case was classified as Grade 4 hepatic encephalopathy according to West Haven criteria and the Hepatic Encephalopathy Scoring Algorithm [5]. Blood and CSF cultures were found to be negative. The diagnosis of hepatic encephalopathy led to the discontinuation of acyclovir on day 3 and ceftriaxone on day 5. The abnormal MRI signals in the brainstem persisted until day 3 (Figure 3) but resolved by day 10 (Figure 4). On day 3, the patient exhibited impaired consciousness with a Glasgow Coma Scale score of E4V1M5. However, by day 10, the patient had regained full consciousness. Blood ammonia levels on day 5 decreased compared to the initial laboratory results, measuring 84 µmol/L. Contrast-enhanced brain MRI on day 3 showed no enhancement of the brainstem lesions. She was ultimately discharged on day 11 in stable condition.

## Discussion

We diagnosed a patient with impaired consciousness and diffuse MRI signal changes in the brainstem as having hepatic encephalopathy caused by primary biliary cholangitis. The brainstem reticular formation regulates consciousness, making the brainstem abnormalities observed on MRI a reasonable explanation for the altered consciousness in this case. The resolution of brainstem signal changes on MRI accompanied by the improvement in consciousness further supports the relationship between these MRI findings and the clinical symptoms. Triphasic waves are typically seen in Grade 2-3 hepatic encephalopathy, and the presence of these waves on day 4 may reflect the transitional phase of improvement in this Grade 4 case [1]. The medial temporal lobes, prone to MRI artifacts, showed no changes over time, supporting the conclusion that hyperintensity in this area was an artifact [10]. Tracking signal alterations over time is crucial for distinguishing artifacts from true pathological changes.

Conditions presenting with abnormal signals localized to the brainstem on brain MRI include vascular lesions, demyelinating inflammatory lesions, infectious diseases including infectious rhombencephalitis, neoplastic lesions, and metabolic encephalopathies such as Wernicke’s encephalopathy and central pontine myelinolysis [11]. In this case, the rapid resolution of MRI findings suggested that vascular lesions were unlikely. The improvement of MRI findings without immunotherapy further indicated that demyelinating inflammatory lesions were unlikely. Additionally, the absence of contrast enhancement or mass effect on MRI did not support a neoplastic etiology. Furthermore, negative blood and CSF cultures, along with CSF analysis showing no signs of infection, ruled out infectious causes. Especially, typical MRI features of metabolic encephalopathies include symmetrical signal changes, hyperintensity on DWI, and reversible imaging findings during the clinical course [12,13]. This case exhibited all of these characteristic findings associated with metabolic encephalopathies, supporting the conclusion that the MRI signal changes were attributable to hepatic encephalopathy. There was no deficiency of vitamin B1 or hyponatremia on laboratory tests, making Wernicke's encephalopathy and central pontine myelinolysis unlikely. Consequently, this case highlights the importance of considering hepatic encephalopathy in the differential diagnosis of patients with MRI signal changes confined to the brainstem.

Brain MRI findings in hepatic encephalopathy can be classified into three distinct signal patterns based on the underlying pathological condition: (1) hyperintensity in the globus pallidus and substantia nigra on T1WI, (2) hyperintensity on DWI with corresponding high ADC values, and (3) hyperintensity on DWI with low ADC values [6,14]. In this case, no signal changes were observed in the globus pallidus or substantia nigra on T1WI, nor were there any high-signal lesions on DWI with high ADC values, both of which are typically associated with chronic changes in hepatic encephalopathy [6]. Conversely, the hyperintensity on DWI with low ADC values, observed in this case, suggests acute cellular edema in hepatic encephalopathy, likely caused by the rapid accumulation of glutamate in astrocytes due to ammonia metabolism. This mechanism is considered a hallmark of acute hepatic encephalopathy [6,14]. In this case, the MRI findings suggest acute cellular edema, which likely contributed to the patient's rapidly impaired consciousness. Similar cases with cellular edema changes on MRI have shown acute onset and rapid progression, further supporting the link between these signal changes and the clinical presentation [15].

Notably, to the best of our knowledge, only three cases of hepatic encephalopathy with brainstem signal changes on MRI have been reported with detailed clinical and imaging findings [14,16,17] (Table 2). The distribution of brainstem signal changes in our case differed from those in the previously reported cases and was the most extensive among them. In contrast, two cases (Case 2 and Case 3) that exhibited widespread signal changes beyond the brainstem, including the cerebrum, had poor prognoses, with either prolonged recovery or fatal outcomes. On the other hand, our case, which showed no signal changes outside the brainstem, and Case 1, which exhibited additional signal changes only in the thalamus, demonstrated rapid clinical improvement. A previous cohort study, which included 98 hepatic encephalopathy patients without detailed clinical and imaging information, indicated that hepatic encephalopathy with MRI signal changes confined to the brainstem tends to exhibit a milder clinical course compared to cases with signal changes involving regions outside the brainstem [18]. This finding aligns with our observations in the literature review that hepatic encephalopathy with MRI signal changes confined to the brainstem or a limited region has a favorable prognosis. However, the previous cohort study did not clearly distinguish between acute and chronic MRI change or discuss the extent of lesion localization [18]. Similarly, prior case reports have not differentiated between acute and chronic changes in MRI findings [14,16,17]. Thus, further prospective studies are warranted to investigate the detailed patterns and distribution of MRI signal changes in hepatic encephalopathy, as well as their correlation with clinical prognosis.

**Table 2:** Case reports of hepatic encephalopathy with abnormal signals in brainstem providing with specific MRI findings as far as we searched [14,16,17] *How FLAIR hyperintense regions appear on DWI and ADC ADC: Apparent diffusion coefficient; AHF: Acute hepatic failure; DWI: Diffusion-weighted imaging; FLAIR: Fluid-attenuated inversion recovery; T2WI: T2-weighted imaging

Study	Case	Age/Sex	Cause	NH_3_ (µmol/L)	Symptoms	MRI				Prognosis	
						Inside of brainstem		Outside of brainstem			
						Hyperintensity areas on FLAIR or T2WI	DWI and ADC*	Hyperintensity areas on FLAIR or T2WI	DWI and ADC*	Outcome	Disease duration
McKinney et al.	1	10/F	AHF after acetaminophen overdose	104	Confusion	Bilateral pontine tegmentum	n.a	Thalami	DWI: high	Survived without sequela	13 days after admission
Filipović Grčić P et al.	2	43/M	Liver cirrhosis	85.6	Asymmetric upper extremity tremor	Crus cerebri, red nuclei	n.a	Corpus callosum, dentate nucleus, periventricular white matter, internal capsule	n.a	Recovered with mild kinetic and postural tremor of the left hand	6 months after liver transplantation
U-King-Im et al. [17]	3	48/M	Fulminant AHF due to acetaminophen overdose	102	Impaired consciousness, abnormal posturing, seizures	Midbrain without crus cerebri	n.a	Bilateral temporoparietal lobes, thalami, insular, cingulate cortices	DWI: high	Died	2 days after admission
Our case	4	75/F	Liver cirrhosis due to primary biliary cholangitis	210	Impaired consciousness	Bilateral tegmentum of midbrain and pons	DWI: high, ADC: low	None	-	Survived without sequela	4 days after admission

This case has a few limitations. First, while continuous EEG monitoring is useful for differentiating epilepsy as a cause of impaired consciousness, our facility was unable to perform this test, and only a single EEG was conducted on day 4. Additionally, polymerase chain reaction (PCR) testing for herpes simplex virus in CSF, which is valuable for excluding herpes encephalitis, was not performed. However, given the multiple findings strongly suggestive of hepatic encephalopathy and the exclusion of other conditions through various tests, we confidently diagnose this case as hepatic encephalopathy.

## Conclusions

We diagnosed a patient with hepatic encephalopathy presenting with diffuse MRI signal changes confined to the brainstem. The MRI features in this case, including symmetrical signal changes, hyperintensity on DWI, and reversible imaging findings during the clinical course, supported the diagnosis of metabolic encephalopathy. Hepatic encephalopathy should be considered in patients with brainstem-limited MRI changes. Early diagnosis and intervention are key for improving outcomes. Additionally, the MRI findings in this case showed hyperintensity on DWI with low ADC values, possibly reflecting acute onset and rapid progression. Moreover, the brainstem-restricted signal changes may indicate a favorable prognosis. However, further research is needed to explore the correlation between MRI findings and prognosis in hepatic encephalopathy.

## References

[1] Wijdicks EF (2016). Hepatic encephalopathy. N Engl J Med.

[2] Sørensen M, Andersen JV, Bjerring PN, Vilstrup H (2024). Hepatic encephalopathy as a result of ammonia-induced increase in GABAergic tone with secondary reduced brain energy metabolism. Metab Brain Dis.

[3] Ridola L, Faccioli J, Nardelli S, Gioia S, Riggio O (2020). Hepatic encephalopathy: diagnosis and management. J Transl Int Med.

[4] Weissenborn K (2019). Hepatic encephalopathy: definition, clinical grading and diagnostic principles. Drugs.

[5] Hassanein TI, Hilsabeck RC, Perry W (2008). Introduction to the Hepatic Encephalopathy Scoring Algorithm (HESA). Dig Dis Sci.

[6] Rovira A, Alonso J, Córdoba J (2008). MR imaging findings in hepatic encephalopathy. AJNR Am J Neuroradiol.

[7] Pujol A, Pujol J, Graus F (1993). Hyperintense globus pallidus on T1-weighted MRI in cirrhotic patients is associated with severity of liver failure. Neurology.

[8] Lim CG, Hahm MH, Lee HJ (2023). Hepatic encephalopathy on magnetic resonance imaging and its uncertain differential diagnoses: a narrative review. J Yeungnam Med Sci.

[9] You H, Duan W, Li S (2023). Guidelines on the diagnosis and management of primary biliary cholangitis (2021). J Clin Transl Hepatol.

[10] Kubota BY, Coan AC, Yasuda CL, Cendes F (2015). T2 hyperintense signal in patients with temporal lobe epilepsy with MRI signs of hippocampal sclerosis and in patients with temporal lobe epilepsy with normal MRI. Epilepsy Behav.

[11] Renard D, Guillamo JS, Ion I, Thouvenot E (2022). Brainstem lesions: MRI review of standard morphological sequences. Acta Neurol Belg.

[12] Sharma P, Eesa M, Scott JN (2009). Toxic and acquired metabolic encephalopathies: MRI appearance. AJR Am J Roentgenol.

[13] Jeon SJ, Choi SS, Kim HY, Yu IK (2021). Acute acquired metabolic encephalopathy based on diffusion MRI. Korean J Radiol.

[14] McKinney AM, Lohman BD, Sarikaya B, Uhlmann E, Spanbauer J, Singewald T, Brace JR (2010). Acute hepatic encephalopathy: diffusion-weighted and fluid-attenuated inversion recovery findings, and correlation with plasma ammonia level and clinical outcome. AJNR Am J Neuroradiol.

[15] Reis E, Coolen T, Lolli V (2020). MRI findings in acute hyperammonemic encephalopathy: three cases of different etiologies. J Belg Soc Radiol.

[16] Filipović Grčić P, Džamonja G, Filipović Grčić A, Dolić K, Matijaca M, Titlić M (2018). Regression of asymmetric upper extremity tremor after liver transplantation in a patient with hepatic encephalopathy: case report. Acta Clin Croat.

[17] U-King-Im JM, Yu E, Bartlett E, Soobrah R, Kucharczyk W (2011). Acute hyperammonemic encephalopathy in adults: imaging findings. AJNR Am J Neuroradiol.

[18] Lim CG, Lee HJ (2024). Pattern clustering of symmetric regional cerebral edema on brain MRI in patients with hepatic encephalopathy. J Korean Soc Radiol.

